# Supplementary material for: Intragenic proviral elements support transcription of defective HIV-1 proviruses
Source: PLoS Pathog. 2021 Dec 28;17(12):e1009982. doi: 10.1371/journal.ppat.1009982 (PMC8746790; doi:10.1371/journal.ppat.1009982)
Supplement: S1 Table — (PDF) [file ppat.1009982.s001.pdf]

S1 Table. Primer and Probe Sequences

IPDA Primers*			
**Assay**	**Primer Name**	**Function**	**Sequence (5' to 3')**
Psi	Psi-Forward	Forward	CAGGACTCGGCTTGCTGAAG
	Psi-Reverse	Reverse	GCACCCATCTCTCTCCTTCTAGC
Env	Env-Forward	Forward	AGTGGTGCAGAGAGAAAAAAGAGC
	Env-Reverse	Reverse	GTCTGGCCTGTACCGTCAGC
RPP30-1	RPP30-1-F	Forward	GATTTGGACCTGCGAGC
	RPP30-1-R	Reverse	GCGGCTGTCTCCACAAG
RPP30-2	RPP30-2-F	Forward	GACACAATGTTTGGTACATGGTTAA
	RPP30-2-R	Reverse	CTTTGCTTTGTATGTTGGCAGAAA

IPDA Probes*		
**Probe Name**	**Fluorophore**	**Sequence (5' to 3')**
Psi	FAM	TTTTGGCGTACTCACCAGT
Env	VIC	CCTTGGGTTCTTGGGA
Env Hypermutation	None	CCTTAGGTTCTTAGGAGC
RPP30-1	HEX	CTGACCTGAAGGCTCT
RPP30-2	FAM	CCATCTCACCAATCATTCTCCTTCCTTC

RT-ddPCR Primers**			
**Assay**	**Primer Name**	**Function**	**Sequence**
Long LTR	Kumar F	Forward	GCCTCAATAAAGCTTGCCTTGA
	Kumar R	Reverse	GGGCGCCACTGCTAGAGA
Nef	F8883-03	Forward	GGTGGGAGCAGTATCTCGAGA
	R9040-10	Reverse	TGTAAGTCATTGGTCTTAAAGGTACCTGAGG

RT-ddPCR Probes**			
**Assay**	**Primer Name**	**Fluorophore**	**Sequence**
Long LTR	Kumar P	FAM	CCAGAGTCACACAACAGACGGGCACA
Nef	P8967-50	FAM	CCAGGCACAAGCAGCATT

*IPDA primer and probe designs first described by Bruner et al.[1]

**RT-ddPCR primer and probe designs first described by Yukl et al.[2]

5' RACE - Gene Specific Nef Primer	
**Primer Name**	**Sequence (5' to 3')**
RACE-Nef-R	GATGGGGTGGGAGCAGTATCTCGAGACC

References

1. Bruner KM, Wang Z, Simonetti FR, Bender AM, Kwon KJ, Sengupta S, et al. A quantitative approach for measuring the reservoir of latent HIV-1 proviruses. [cited 2019 Dec 13]; Available from: https://doi.org/10.1038/s41586-019-0898-8

2. Yukl SA, Kaiser P, Kim P, Telwatte S, Joshi SK, Vu M, et al. HIV latency in isolated patient CD4+ T cells may be due to blocks in HIV transcriptional elongation, completion, and splicing. Sci Transl Med [Internet]. 2018 Feb 28 [cited 2021 Sep 15];10(430):9927. Available from: /pmc/articles/PMC5959841/
