# Supplementary material for: Intragenic proviral elements support transcription of defective HIV-1 proviruses
Source: PLoS Pathog. 2021 Dec 28;17(12):e1009982. doi: 10.1371/journal.ppat.1009982 (PMC8746790; doi:10.1371/journal.ppat.1009982)
Supplement: S2 Table — (PDF) [file ppat.1009982.s002.pdf]

S2 Table. Participant Details - People Living with HIV on Antiretroviral Therapy

Participant	Age	Sex	Race	Ethnicity	CD4 at enrollment	Nadir CD4	Years on ART
A014	66	Male	White	Not Hispanic or Latino	1523	508	12.4
A033	60	Female	Black / African American	Not Hispanic or Latino	394	394	6.4
A042	68	Female	Black / African American	Not Hispanic or Latino	494	619	13.2
A044	54	Female	White	Not Hispanic or Latino	932	192	7.2
A058	64	Male	Black / African American	Not Hispanic or Latino	567	567	6.7
A061	59	Female	Black / African American	Not Hispanic or Latino	625	543	12.5
W102	75	Male	Black / African American	Not Hispanic or Latino		182	5.1
W124	67	Male	White	Not Hispanic or Latino	810	284	4.4
W143	33	Male	Other	Hispanic/Latino	471	270	3.2
X101	55	Female	Black / African American	Not Hispanic or Latino	414	259	2.7
X104	51	Male	Black / African American	Not Hispanic or Latino	1055	607	3.3
