# Supplementary material for: Intragenic proviral elements support transcription of defective HIV-1 proviruses
Source: PLoS Pathog. 2021 Dec 28;17(12):e1009982. doi: 10.1371/journal.ppat.1009982 (PMC8746790; doi:10.1371/journal.ppat.1009982)
Supplement: S5 Table — (PDF) [file ppat.1009982.s005.pdf]

S5 Table. Human Transcription Factor Binding Events Identified by Yeast-1-Hybrid Assay with HIV-1 Tile Sequences

Transcription Factor	Family	HIV Tile Sequence
Iroquois homeobox protein 5	HD	HIV1-2, HIV1-11, HIV1-14, HIV1-15
Nuclear factor 1 A-type	MH1	HIV1-2
Iroquois homeobox protein 6	HD	HIV1-2, HIV1-11, HIV1-15
Homeobox protein Hox-C8	HD	HIV1-2
SRY-Box Transcription Factor 14	HMG	HIV1-2, HIV1-16
Nuclear factor 1 C-type	MH1	HIV1-2
PLAG1 Like Zinc Finger 1	ZF-C2H2	HIV1-2
Homeobox protein Hox-A6	HD-HOX	HIV1-2, HIV1-16
Transcription factor AP-2 beta	AP-2	HIV1-10, HIV1-14, HIV1-15
Grainyhead Like Transcription Factor 2	CP2	HIV1-10, HIV1-14, HIV1-16
Forkhead box protein H1	WH - FH	HIV1-11
Twist Family BHLH Transcription Factor 1	bHLH	HIV1-11
Class B basic helix-loop-helix protein 8	bHLH	HIV1-11
Runt-related transcription factor 1	RUNT	HIV1-11
Runt-related transcription factor 3	RUNT	HIV1-11
Zinc finger protein basonuclin-1	ZF - C2H2	HIV1-11
Zinc Finger Protein 710	ZF - C2H2	HIV1-11, HIV1-16
Musculin	bHLH	HIV1-11
Transcription factor 21	bHLH	HIV1-11
Transcription factor EC	bHLH	HIV1-11
MYC Associated Factor X	bHLH	HIV1-11
Atonal BHLH Transcription Factor 1	bHLH	HIV1-11, HIV1-14
T-Box Transcription Factor 22	T-BOX	HIV1-11
POU Class 4 Homeobox 3	HD - POU	HIV1-11
Myogenic Factor 6	bHLH	HIV1-11

Transcription Factor	Family	HIV Tile Sequence
Zinc Finger And BTB Domain Containing 10	ZF - C2H2	HIV1-11, HIV1-14
Glial Cells Missing Transcription Factor 1	GCM	HIV1-11
ETS2 Repressor Factor	WH - ETS	HIV1-14
CAMP Responsive Element Binding Protein 5	bZIP	HIV1-14
Zic Family Member 3	ZF - C2H2	HIV1-14
Zic Family Member 1	ZF - C2H2	HIV1-14
E74 Like ETS Transcription Factor 2	WH - ETS	HIV1-14, HIV1-16
Transcription Factor AP-2 Alpha	AP-2	HIV1-14
T Cell Leukemia Homeobox 3	HD	HIV1-14
Haematopoietically-expressed homeobox protein	HD	HIV1-14, HIV1-16
Early B-cell factor 3	IPT/TIG	HIV1-15, HIV1-16
Forkhead Box J2	WH - FH	HIV1-16
Krüppel Like Factor 4	ZF - C2H2	HIV1-16
SIX Homeobox 6	HD - SIX	HIV1-16
Spi-1 Proto-Oncogene	WH - ETS	HIV1-16
REL Proto-Oncogene, NF-KB Subunit	IPT/TIG, p53	HIV1-16
Early B-cell factor 1	IPT/TIG	HIV1-16
Nuclear Receptor Subfamily 2 Group F Member 2	ZF - NHR	HIV1-16
