# Supplementary figures and images for: Intragenic proviral elements support transcription of defective HIV-1 proviruses
Source: PLoS Pathog. 2021 Dec 28;17(12):e1009982. doi: 10.1371/journal.ppat.1009982 (PMC8746790; doi:10.1371/journal.ppat.1009982)

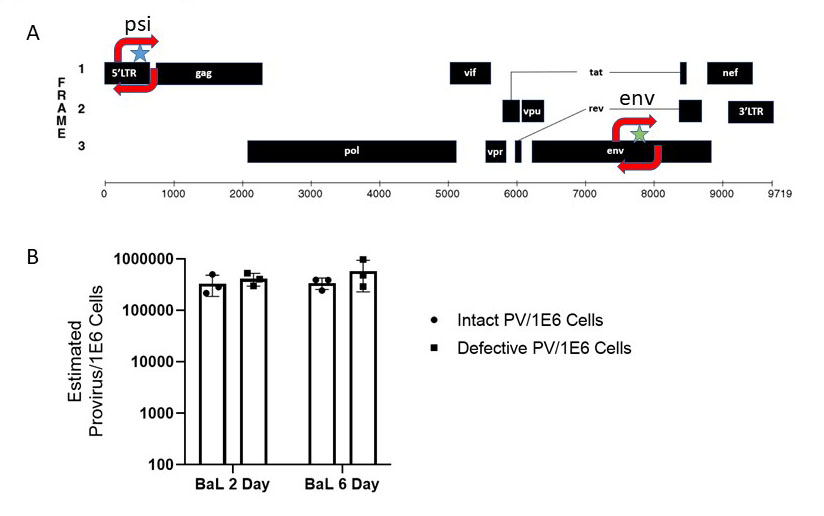

Supplement: S1 Fig — (A) Schematic of primer (arrows) and probe (stars) binding sites used for IPDA. For details see methods. (B) IPDA time course data for HIV-1 NL4-3-BaL infected MDMs. MDMs differentiated from 3 separate donors were infected as described in Methods and incubated for either 2 or 6 days before DNA isolation. IPDA was used to estimate intact and defective provirus frequencies per 1x10^6^ cells at each time point. Day 2 and Day 6 intact and defective provirus estimates were not statistically different when analyzed by Two-Sample T Test. (TIF) [file ppat.1009982.s007.tif]

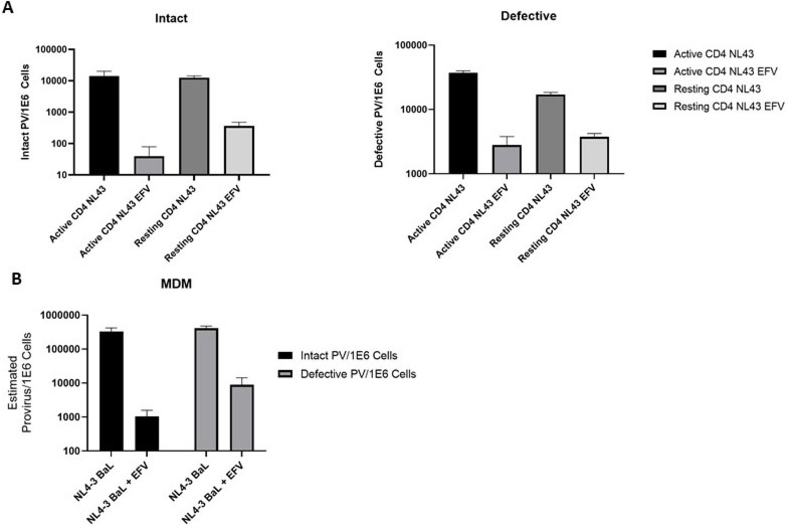

Supplement: S2 Fig — (A) IPDA data for EFV pretreated CD4+ T cells infected with HIV-1NL4-3. For CD4+ T cells, EFV was added at a concentration of 10 μM 30 minutes prior to spinoculation with HIV-1NL4-3. Infection was limited to a single round by addition of the viral protease inhibitor Saquinavir 30 minutes after spinoculation. Resting and activated CD4+ T cells were participant matched. Data are from 3 separate donors. (B) IPDA data for EFV pretreated MDMs infected with HIV-1NL4-3-BaL. For MDMs, EFV was added at a concentration of 10 μM 24-hours prior to addition of HIV-1NL4-3-BaL to the cell cultures. Data are from 3 separate donors. (TIF) [file ppat.1009982.s008.tif]

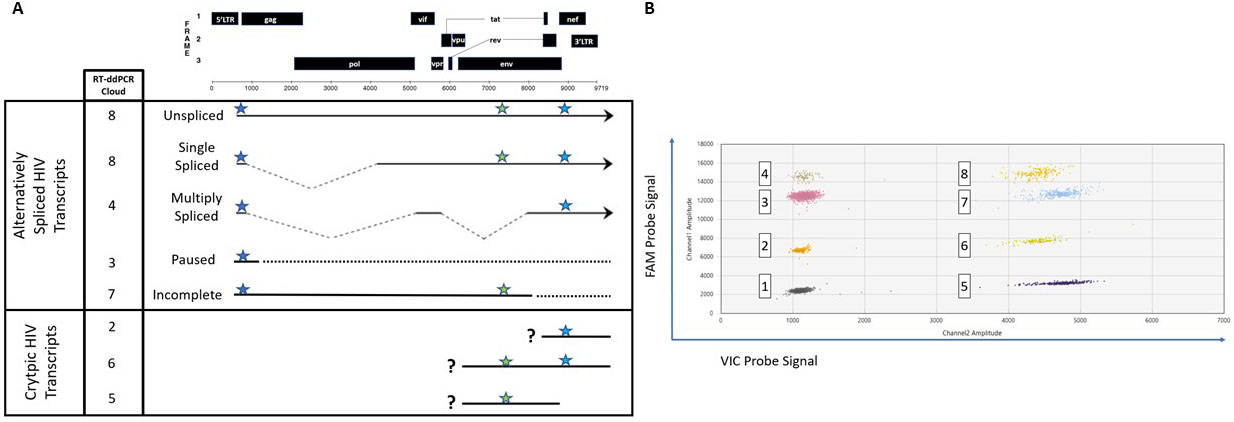

Supplement: S3 Fig — (A) Schematic of RT-ddPCR probe binding sites aligned to the HIV genome. Traditional HIV transcripts are labeled with population numbers that correlate to the droplet populations shown in (B). Putative cryptic HIV transcripts lacking 5’UTR sequence are labeled as populations 2, 6, and 5. Population 1 represents empty droplets or HIV transcripts which lack all probed sequence sites. (B) Representative data acquired during multiplex RT-ddPCR. Data shown reflects the following multiplexed probe reactions: 1X concentration LTR probe reaction (y-axis, FAM), 0.5X Nef probe reaction (y-axis, FAM), and 1X Env probe reaction (x-axis, VIC). The multiplexed assay detected 8 distinct populations which were manually gated as reflected by different droplet colors and numbers above. Numbers represent droplets harboring the following distinct transcriptional species: (1) Empty droplets, (2) Nef Only Transcripts, (3) LTR Only Transcripts, (4) LTR+Nef Transcripts, (5) Env Only Transcripts, (6) Env+Nef Transcripts, (7) LTR+Env Transcripts, (8) LTR+Env+Nef Transcripts. (TIF) [file ppat.1009982.s009.tif]

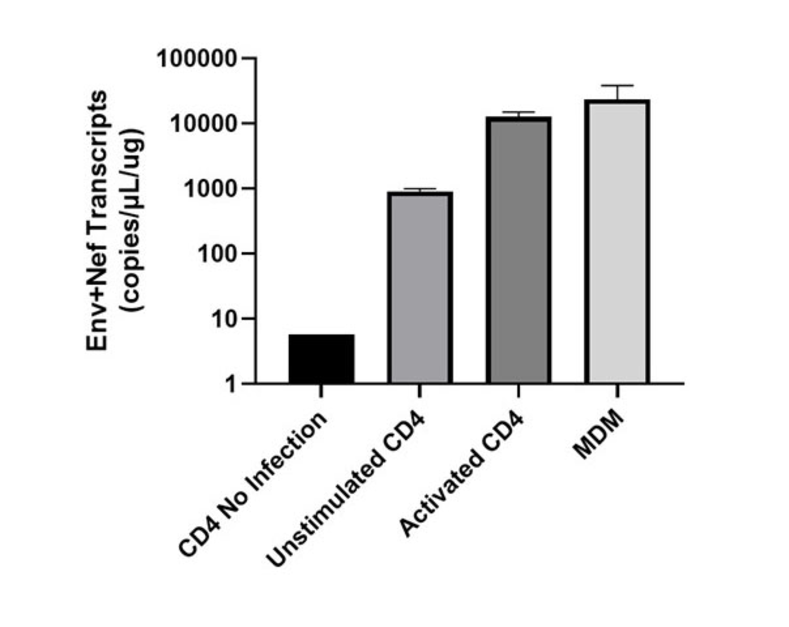

Supplement: S4 Fig — RT was performed as described in Methods using a poly d(T) primer for reverse transcription of 500ng of RNA from either HIV-1NL4-3-BaL infected MDMs or HIV-1NL4-3 infected CD4+ T cells which were unstimulated or activated for 72 hours using anti-CD3/CD28 beads. No Infection negative control sample is representative of N = 1. All remaining data are representative of three separate infections. CD4+ T cell data are donor matched between unstimulated and activated cells. (TIF) [file ppat.1009982.s010.tif]
