# Supplementary material for: Intragenic proviral elements support transcription of defective HIV-1 proviruses
Source: PLoS Pathog. 2021 Dec 28;17(12):e1009982. doi: 10.1371/journal.ppat.1009982 (PMC8746790; doi:10.1371/journal.ppat.1009982)
Supplement: Attachment — Submitted filename: Revision Cover Kuniholm et al.doc [file ppat.1009982.s011.doc]

2 December 2021

Dear Editorial Board of *PLOS Pathogens*,

Thank you for the handling of our manuscript by Kuniholm et al. titled “Intragenic proviral elements support transcription of defective HIV-1 proviruses.” The editor invited us to resubmit our manuscript after addressing revisions suggested by the reviewers. In general, the reviewers were positive and emphasized that the manuscript was “well-written and clearly presented” and presents “novel findings”, providing “some of the best data” regarding the function of intragenic elements and transcription of defective HIV-1 provirus. The reviewers did have some criticisms regarding interpretation of IPDA data, requested some experimental details, and suggested formatting for data presentation. Furthermore, the reviewers requested additional information about transcription factor binding site motifs and the organization of putative transcriptional elements. Accordingly, we have provided additional data which includes annotated 5’ RACE PCR sequences and a new figure to illustrate the proximity of aberrant HIV-1 transcript initiation sites to start codons and TATA box sequences in the HIV-1 *env* gene (Figure 4). We have also provided information regarding identified transcription factor binding motifs and their locations within the identified intragenic element that spans regions 14 and 15 of the tiled HIV-1 genome (Figure 5 and Supplemental Table 6). All editorial points have been addressed and track changes were used in the “marked” revised manuscript to clearly identify these edits. Below we address the specific criticisms in detail.

**Responses to Reviewer 1.**

**Major Comments:**

None

**Minor Comments:**

1. The reviewer suggested that the transcriptional start site of HIV-1 transcripts lacking 5’ sequence could be identified using our HIV-1 Luciferase reporter constructs used to screen for promoter activity. We have mapped HIV-1 transcriptional start sites to an approximately 200 base pair region in the *env* gene, suggesting that there is not a consensus start codon for all aberrant transcripts detected but, rather, a cluster of potential alternative transcriptional start sites. We have added a figure which aligns 5’ RACE sequences to the *env* gene of HIV-1 and highlighted start codons and TATA box sequences to better indicate where these RNAs initiate relative to the start codons in this transcriptionally active region of the *env* gene. We have also provided the 5’ RACE sequences of transcripts lacking 5’ LTR sequence and indicated their starting nucleotide in supplemental table 3. The predicted binding motifs of transcription factors identified in the yeast-1-hybrid assay for regions 14 and 15 have also been included in the revised manuscript (Supplemental Table 6).
2. The reviewer felt that the use of the term pauciclonal was self-contradictory when referring to the accumulation of defective HIV proviruses over time. We have removed this descriptor to avoid any confusion.

**Responses to Reviewer 2.**

**Major Comments:**

1. The reviewer correctly pointed out that HIV-1 proviruses that are negative for the *psi* element specific ddPCR probe represent proviruses with deleted and mutated 5’ LTR promoter sequence and that we should not assume these products harbor 5’ deletions. We have revised our text to reflect that we are detecting defective proviruses and not deleted proviruses.
2. The reviewer requested that 5’ RACE PCR sequences be reported and aligned with the NL4-3 *env* gene with start codons and TATA box sequences indicated. We have now included this information in Figure 4C which aligns all 5’ sequence-deficient RACE PCR sequences with the NL4-3 *env* gene. We have indicated in-frame start codons, TATA box sequences, and out-of-frame ATG sequences in this region. We have also provided the 5’ RACE sequences of transcripts lacking 5’ UTR sequences and indicated their starting nucleotide in Supplemental Table 3.
3. The reviewer asked where the second *Stu*I restriction enzyme site was which enabled deletion of the 6,825bp of the NL4-3 genome. There is a second *Stu*I site in cellular genomic sequences 282 bp 5’ to NL4-3 sequences. The construct was confirmed by DNA sequencing. These details have been added to the revised methods.
4. The reviewer requested that we provide the sequence and motif specificity of transcription factors which bind in the region where 5’ sequence deficient transcripts initiate. In the revised manuscript, we have included Supplemental Table 6 which provides this information.

**Minor Comments:**

1. We have corrected the title of Figure 2 from “CD4+ T cells and MDMs express aberrant RNAs.” to read “CD4+ T cells and MDMs express aberrant HIV-1 RNAs.”
2. We have edited the text to use italic and all lower case when describing HIV RNAs.
3. We have edited Figure 5, Supplemental Figure 1, and Supplemental Figure 3 to highlight the reading frames of the HIV genome.
4. We have italicized the first three letters of the restriction enzyme used for the generation of our NL4-3 deletion construct (*Stu*I).
5. We have removed any use of the word “patient” when referring to people living with HIV. We have updated the term “patient matched” to “participant matched.”
6. All figures reporting of HIV DNA and RNA copy numbers (Figure 5B, Supplemental Figure 1B, Supplemental Figure 2) have been updated to log-scale.
7. Table outlines of Supplemental Table 1 have been updated for consistency.

**Responses to Reviewer 3**

**Major Comments:**

1. The reviewer stated that a substantial proportion of activated cells may not be long-lived during HIV infection and indicated that “it would be very useful to perform serial studies of the cell populations over time, to ascertain if the predominant intact provirus is found largely in cells fated for short half-lives.” The dynamics of the proviral landscape during different stages of HIV infection, including acute and chronic HIV infection, is a topic of great interest in the field and the lab, and as suggested by the reviewer reflects intrinsic properties of the infected cell, the impact of HIV on the cell, provirus expression and immune surveillance and clearance of HIV-1 infected cells. We are interested in expanding our studies beyond in vitro acute infections which do not recapitulate immune selection over time and examining intact and defective provirus expression in different longitudinal PLWH cohorts; however, these studies are beyond the scope of this current manuscript which demonstrates that cryptic RNAs can be generated from defective proviruses.
2. The reviewer requested an estimation of the frequency of the aberrant HIV-1 transcripts detected per million cells. We have presented our results as relative concentrations of UTR containing transcripts and 5’ UTR-deficient *env* transcripts after multiplex RT-ddPCR as copies/uL, as reported by the droplet digital PCR data acquisition software, per mg of RNA used in the RT reaction and both of these RNAs were detected in PBMC samples from PLWH in relatively similar concentrations. The comparison of relative RNA expression detected is now included in the text where we discuss these results. The experiments were performed on bulk cell populations, so we are not able to measure the contribution of transcription by individual infected cells.
3. The reviewer suggested several experiments utilizing samples from PLWH that would allow us to examine the correlation between these cryptic RNAs and markers of inflammation. We do speculate in the discussion on the potential functional impact of these RNAs on HIV associated diseases and these experiments are currently ongoing in our group. However, our results examining whether these RNAs and proteins influence immune function are too preliminary and we need to fully understand the mechanism prior to including these data.

**Minor Comments:**

1. We have updated references as stated by the reviewer.

Sincerely,

Andrew J. Henderson, Ph.D.

Associate Professor of Medicine and Microbiology

hender@bu.edu

Tel 617-414-5240
